# Towards comprehensive annotation of *Drosophila melanogaster* enzymes in FlyBase

**DOI:** 10.1093/database/bay144

**Published:** 2019-01-23

**Authors:** Phani V Garapati, Jingyao Zhang, Alix J Rey, Steven J Marygold

**Affiliations:** Department of Physiology, Development and Neuroscience, University of Cambridge, Downing Street, Cambridge, CB2 3DY, UK

## Abstract

The catalytic activities of enzymes can be described using Gene Ontology (GO) terms and Enzyme Commission (EC) numbers. These annotations are available from numerous biological databases and are routinely accessed by researchers and bioinformaticians to direct their work. However, enzyme data may not be congruent between different resources, while the origin, quality and genomic coverage of these data within any one resource are often unclear. GO/EC annotations are assigned either manually by expert curators or inferred computationally, and there is potential for errors in both types of annotation. If such errors remain unchecked, false positive annotations may be propagated across multiple resources, significantly degrading the quality and usefulness of these data. Similarly, the absence of annotations (false negatives) from any one resource can lead to incorrect inferences or conclusions. We are systematically reviewing and enhancing the functional annotation of the enzymes of *Drosophila melanogaster*, focusing on improvements within the FlyBase (www.flybase.org) database. We have reviewed four major enzyme groups to date: oxidoreductases, lyases, isomerases and ligases. Herein, we describe our review workflow, the improvement in the quality and coverage of enzyme annotations within FlyBase and the wider impact of our work on other related databases.

## Introduction

Enzymes are biocatalysts that greatly enhance the rate of specific chemical reactions without being consumed in the process—without enzymatic catalysis, most reactions would be too slow to sustain life. Enzymes are at the heart of all cellular processes. Metabolic pathways are built from enzymes acting sequentially to perform chemical conversions such as the breakdown of glucose to lactate or the synthesis of the neurotransmitter dopamine from the amino acid l-phenylalanine. Other enzymes, including protein kinases and phosphatases, play crucial roles in signaling networks that regulate a wide range of essential cellular activities, including proliferation, differentiation, survival and apoptosis (1).

Loss or malfunction of a single critical enzyme can lead to multiple genetic disorders including diabetes, hypertension and cancers (2). The classical and prominent example is phenylketonuria, an inborn error of metabolism, caused by mutations in the phenylalanine hydroxylase enzyme (3). Another example is the RAS GTPase: mutated H-RAS, N-RAS or K-RAS is found in >20% of all human cancers and are among the most important drug targets in oncology (4). Enzymes are also used in medical diagnosis of various disease states. For example, phosphoglycerate dehydrogenase is used as a marker for breast cancer and melanoma (5). Thus, determining and cataloging the biological activities of enzymes not only helps us better understand metabolic systems but also provides important insights on disease mechanisms, detection and potential therapies.

The enzymatic activity of a gene product can be annotated with Enzyme Commission (EC) numbers and Gene Ontology (GO) identifiers. EC numbers define the overall chemical transformation mediated by a particular enzyme, encapsulated as a four-number code (6). The first number corresponds to one of seven different classes according to the type of chemistry being carried out; the second and third numbers correspond to the chemical bond acted on (subclasses) and reaction (sub-subclasses), respectively; and the last number defines substrate specificity. For example, alanine racemase is an isomerase (EC 5), in particular a racemase (EC 5.1) that acts on the amino acid (EC 5.1.1) alanine (EC 5.1.1.1). EC annotations are prevalent in metabolic pathway-oriented databases such as MetaCyc/BioCyc (7) and KEGG (8) and are also used in UniProtKB (9), NCBI Gene (10) and some model organism databases (MODs).

The GO is the most widely used controlled vocabulary describing gene product function (11). GO annotations unify functional descriptions across all the major MODs, UniProtKB and other biological databases. Unlike EC codes, GO terms are assigned to genes/gene products as part of a fuller annotation that includes the source of the assertion and an `evidence code’ (12) to describe how the annotation to a particular term is supported. GO annotations may be made based on:

- experimental data,
- computational analyses of gene/gene product sequences,
- author statements or
- inferences from electronic (automated) pipelines, such as the presence of protein domains via ‘InterPro2GO’ mapping (Table 1).

Importantly, catalytic activity terms in the GO are cross-referenced to equivalent EC numbers. For example, EC 5.1.1.1 is a cross-reference for the GO term `alanine racemase activity’ (GO:0008784).

**Table 1:** Evidence types used in the GO annotations relevant to this study

**Evidence code (abbreviation)**	**Evidence and conclusion ontology ID**
**Experimental evidence codes**	
Inferred from direct assay (IDA)	ECO:0000314
Inferred from mutant phenotype (IMP)	ECO:0000315
Inferred from genetic interaction (IGI)	ECO:0000316
**Computational analysis evidence codes**	
Inferred from sequence or structural	ECO:0000250
similarity (ISS)^a^	
Inferred from biological aspect of ancestor (IBA)	ECO:0000318
**Author statement evidence codes**	
Traceable author statement (TAS)	ECO:0000304
Non-traceable author statement (NAS)	ECO:0000303
**Electronic annotation evidence code**	
Inferred from electronic annotation (IEA)^b^	ECO:0000501
InterPro2GO mapping	
UniProtKB EC2GO mapping	
UniProtKB Keywords2GO mapping	
UniProtKB UniRule mapping	

a ^a^More specific evidence codes exist as children of ISS but are not shown here.

b ^b^IEA is used as the evidence code for all of the given automated mapping pipelines.

The fruit fly, *Drosophila melanogaster*, has been used as a model system to study enzyme function for over a century. Many classical eye color mutations were later found to disrupt enzymes (13) and experiments in the 1930s and 1940s by Beadle and colleagues helped establish the connection between genes, enzymes and metabolic pathways [reviewed in (14)]. Later studies in the 1970s and 1980s combined fly genetics with biochemistry, leading to seminal discoveries on pyrimidine biosynthesis (15), purine metabolism (16) and alcohol dehydrogenase (17), among others. Today, the availability of a complete, well-annotated genome, together with an increasing array of powerful genetic tools, makes enzymology research in *D. melanogaster* even more attractive. Moreover, the high degree of evolutionary conservation of many enzymes, particularly those involved in metabolism, means that knowledge established in one organism may be transferred to another. Thus, the function of many uncharacterized fly enzymes can be reasonably inferred via orthology, while novel enzymatic research in flies can provide insights with high clinical relevance (18).

FlyBase (www.flybase.org) is the authoritative source for genetic, genomic and functional information on *D. melanogaster* and related fly species (19). There are 13 932 protein-coding genes annotated in the latest FlyBase release (FB2018_05), of which 4153 (30%) are annotated with an enzymatic activity from the GO. The majority of these annotations have never been systematically reviewed or compared to data available from other sources, meaning that enzymatic data presented within FlyBase (and third-party sites) may be incomplete or inaccurate. In order to assess and address these shortcomings, we have begun to catalog all known and predicted *D. melanogaster* enzymes, making use of all available data sources. Our primary aim is to evaluate and enhance the overall quality of GO annotations for *D. melanogaster* enzymes and to improve access to validated enzyme lists within FlyBase. In so doing, we are also improving the general quality of enzyme annotation available at other popular resources, including QuickGO, UniProtKB and GenBank/NCBI.

## Materials and methods

### *D. melanogaster* GO annotations

*D. melanogaster* GO annotations were obtained from FlyBase (http://flybase.org/) by using the `GO’ tab of the QuickSearch tool to find the `Term Report’ for a given GO term and thence obtain a `Hitlist’ of *D. melanogaster* genes annotated with that term or any of its children. Hits were exported to the `Batch Download’ tool where the associated GO annotations were downloaded as a TSV file.

*D. melanogaster* GO annotations were obtained from QuickGO (https://www.ebi.ac.uk/QuickGO/) by searching for a given GO term and filtering the associated list of annotations on *D. melanogaster* (Taxon = 7277). Initially, all sources of annotation and all evidence codes were considered, but we later refined this to exclude electronic annotations computed by UniProtKB (Table 1; explained in the Results). Selected annotations were downloaded in TSV format. The UniProtKB accessions associated with each annotation were converted to FlyBase gene identifiers (FBgn) using the UniProt `Retrieve/ID mapping’ tool (https://www.uniprot.org/uploadlists/); unconverted accessions were mapped manually by searching FlyBase with the given identifiers and/or BLAST.

### *D. melanogaster* EC annotations

*D. melanogaster* EC annotations were obtained from NCBI Gene using their advanced search interface (https://www.ncbi.nlm.nih.gov/gene/advanced) to search the `EC/RN Number’ field with all relevant EC numbers for a given class (using the `show index list’ option) and joining this with a search for `Taxonomy ID’ = 7227. The results were filtered on `Status’ = Current and downloaded as an XML file, which was then parsed to obtain FBgn and associated EC numbers.

*D. melanogaster* EC annotations were obtained from UniProtKB using their advanced search interface (https://www.uniprot.org/) to search the `Function’→`Enzyme classification’ field with the relevant EC numbers for a given class [e.g. `Ligases (6.-.-.-)’] and joining this with a search for `Taxonomy’ = 7227. Initially, both reviewed (Swiss-Prot) and unreviewed (TrEMBL) accessions were considered, but we later restricted this to Swiss-Prot accessions only (explained in the Results). The columns in the search results were edited to include the EC number and the FBgn ID and the results downloaded in TSV format. Any UniProtKB accessions that were not automatically mapped to FBgn identifiers were mapped manually by searching FlyBase with the given identifiers and/or BLAST.

*D. melanogaster* EC annotations were retrieved from KEGG by accessing the BRITE hierarchy of enzymes (https://www.kegg.jp/kegg-bin/get_htext?ko01000.keg) and selecting `*Drosophila melanogaster*’. The `htext’ file was downloaded and parsed to obtain *D. melanogaster* annotation IDs (CG numbers) corresponding to each enzyme class. CG numbers were converted to FBgn IDs using the FlyBase ID converter tool (http://flybase.org/convert/id).

### Identification of human enzymes and their *D. melanogaster* orthologs

Well-characterized human enzymes of a given class were identified via GO annotations (by querying QuickGO, restricted to manual annotations) and EC annotations (by querying UniProtKB/Swiss-Prot only) following the protocols given above. The resulting UniProt accessions were made unique and submitted to the DRSC Integrative Ortholog Prediction Tool (DIOPT) (https://www.flyrnai.org/cgi-bin/DRSC_orthologs.pl), with `*Homo sapiens*’ set as the input species and `*Drosophila melanogaster*’ set as the output species. The `Exclude low scores (score > 2)’ filter was applied to remove genes where only one or two of the 14 + individual orthology algorithms support a given orthologous relationship. The returned *D. melanogaster* orthologs and associated data were downloaded as an Excel file.

### Integration and assessment of annotations

GO annotation data for a given enzyme class/subclass were assembled and organized using Google sheets and assessed at gene level. Each gene was then classified as having `high’, `medium’ or `low’ support for encoding an enzyme with the given activity based on the quantity and evidence type of their associated GO annotations (Table 1). `High confidence’ genes have either (i) multiple positive GO annotations with an experimental evidence code for a given activity or (ii) only one such GO annotation that is supported by at least one other positive annotation with a computational analysis or electronic (automated) annotation evidence code. `Medium confidence’ genes lack any annotations with an experimental evidence code but have multiple, independent positive GO annotations with a computational analysis and/or electronic annotation evidence code—typically, IBA and IEA (InterPro2GO) evidence. Any remaining genes are classed as `low confidence’. These include genes with a single supporting GO annotation for the given activity, genes annotated only with `author statement’ evidence codes and genes annotated with apparently conflicting annotations [e.g. two different enzymatic activities or a combination of positive and negative (using the `NOT’ qualifier) statements]. Annotations for low-confidence genes were investigated and either verified or corrected/disputed (see Results).

Enzyme-encoding genes identified via EC annotations or orthology to human enzymes were integrated into the GO-based spreadsheet. Novel genes were investigated - either the genes were verified as encoding enzymes of the given activity or their underlying annotations were corrected/disputed (see Results).

### Venn diagram generation

The Venn diagrams were produced using Venny 2.1 (http://bioinfogp.cnb.csic.es/tools/venny/).

### Calculation of precision, recall and F1 scores

Having determined the number of true positives (TP), false positives (FP) and false negatives (FN) for the *D. melanogaster* ligases found via different methods, the precision (P), recall (R) and F1 scores were determined according to the following formulae: P = TP/(TP + FP), R = TP/(TP + FN) and F1 = 2*(P*R)/(P + R).

## Results

### Data sources

*D. melanogaster* enzymes may be identified directly through existing GO or EC annotations present in several different biological databases, including FlyBase (19), QuickGO (20), UniProtKB (9), NCBI Gene (10) and KEGG (8). In addition, we considered that an indirect search for *D. melanogaster* enzymes through orthology to well-characterized human enzymes could reveal novel candidate enzymes. It was not obvious before we started this project which of these approaches would generate the `best’ list of *D. melanogaster* enzymes or how much overlap or discrepancy there would be across these different annotation methods and databases.

We initially downloaded GO annotations from FlyBase because it is the authoritative source of *D. melanogaster* GO annotations, and it is this annotation set that appears in the GO Consortium’s AmiGO web browser (11). GO data in FlyBase comprises high-quality, manually curated annotations supplemented with annotations from a restricted set of electronic annotations (21). Notably, FlyBase does not incorporate automated GO annotations computed by UniProtKB (22, 23), while InterPro2GO mappings (24) are filtered to remove those redundant with manual annotations (Table 1). The FlyBase website is updated approximately every 2 months, which is helpful to provide defined releases on which to base data analyses/comparisons, but does mean that FlyBase does not necessarily show the most up-to-date GO annotations or use the latest version of the GO itself. As we (i) wished to access the broadest range of GO annotations for our study, (ii) use the most up-to-date versions of the GO and GO annotations and (iii) wished to use certain filter and download tools available at a specialist database, we switched to using QuickGO (20) as our source of *D. melanogaster* GO annotations. QuickGO is powered by the GOA database, which is populated by manual and electronic (automated) annotations provided by UniProt, InterPro, GO_Central, specialist annotation groups and MODs, including FlyBase (23). Ultimately, we determined that the automated GO annotations computed by UniProtKB (Table 1) did not add useful information—annotations generated by EC2GO mapping are somewhat circular as the majority are derived from existing GO annotations in FlyBase via a GO-to-EC mapping pipeline (described below), while annotations made via Keywords2GO or `UniRule’ mapping were found at best to be redundant with other annotations or at worst to be wrong (see the ligase case study below). For these reasons, and to increase the efficiency of our workflow, electronic GO annotations from UniProtKB were excluded from our QuickGO query. In contrast, the electronic annotations generated by InterPro2GO mapping (Table 1) frequently added value and/or filled gaps, and this pipeline was retained.

*D. melanogaster* EC annotations were initially obtained from NCBI Gene (10) as this source appeared to have the greatest coverage of annotations across the genome. However, these EC numbers are derived from the FlyBase GO annotation set at the time of our annual submission of the *D. melanogaster* genome annotation to GenBank and are therefore redundant and out of sync with current FlyBase GO annotations. These annotations were not considered further in our analysis. Instead we turned to UniProtKB (9) as a source of *D. melanogaster* EC annotations. Here, EC numbers are curated manually into the description line of reviewed (Swiss-Prot) entries and added computationally to unreviewed (TrEMBL) entries. The former category proved to be a high-quality annotation set somewhat independent of the main GO annotation set. In contrast, the TrEMBL EC annotations were found to be almost entirely redundant with those obtained from NCBI Gene, as the majority of these data are ultimately coming from the same source (i.e. the GenBank submission made by FlyBase). For these reasons, and to streamline our workflow, EC annotations retrieved from UniProtKB were restricted to the reviewed/Swiss-Prot accessions. Finally, KEGG (8) was identified as another independent source of *D. melanogaster* EC annotations. In this database, genes are linked to KEGG Orthology (KO) identifiers and each KO is associated with manually selected EC numbers as part of their definition (25).

Lists of well-characterized human enzymes were generated based on manual GO annotations in QuickGO and EC annotations within UniProtKB/Swiss-Prot records. These were submitted to the DIOPT orthology tool (26) to produce a set of candidate *D. melanogaster* orthologs supported by at least three independent orthology algorithms (i.e. DIOPT score > 2). We wished only to pursue cases of unambiguous orthology. Thus, individual orthology pairs were inspected manually to remove cases with relatively low DIOPT scores or where the relevant enzymatic domain was lacking. In practice, this meant that orthology calls with a DIOPT score of 5 or greater were usually retained, with the majority of retained cases scoring >10.

### Workflow

**Figure 1:**
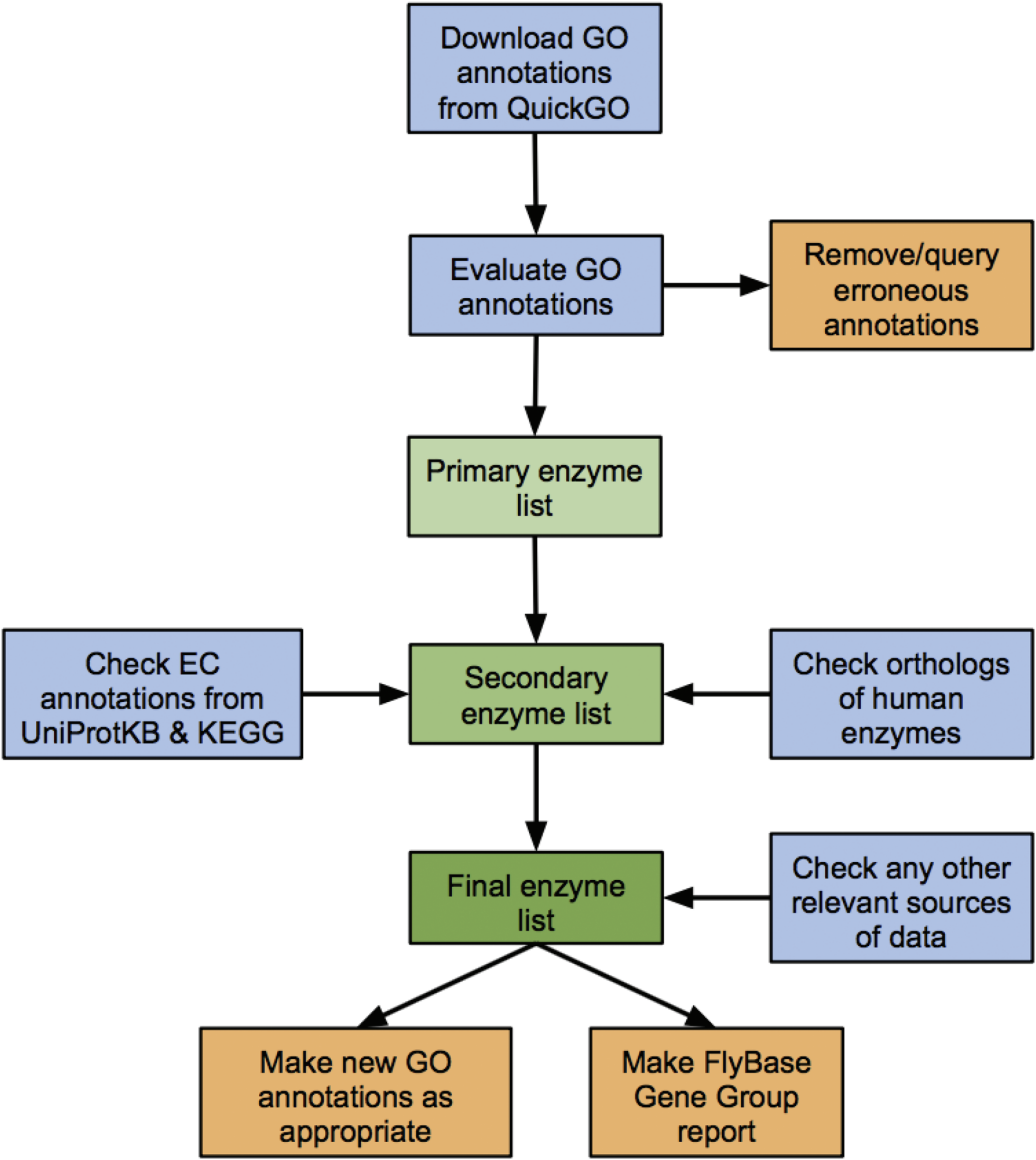
Workflow of the enzyme annotation review process. Blue boxes: download/evaluation steps; green boxes: assembly of the verified list of enzymes; orange boxes: tangible outputs.

We developed a standardized workflow to identify, collate and critically assess all known and predicted enzymes of *D. melanogaster*, based on the data sources discussed above (Figure 1). While our approach focuses on GO annotation, it also considers EC annotations, orthology-based predictions and any other relevant data sources to cover potential gaps in the GO data set.

*D. melanogaster* GO annotations corresponding to a particular enzyme class (e.g. `ligase activity’ or `isomerase activity’) are downloaded from QuickGO and candidates ranked as having `high’, `medium’ or `low’ support for encoding an enzyme with the given activity based on the number and evidence type of their associated GO annotations (see Materials and methods). Genes within the high- and medium-confidence sets are passed to the final list of validated enzymes, while annotations for genes within the low-confidence set are scrutinized further, checking the original source and method of annotation. Any erroneous annotations are corrected/disputed, while tickets are made within the appropriate tracker to report any systematic bugs. Reasons for these false positives include human error in manual annotations and incorrect inferences in computational analysis or electronic annotation pipelines (discussed further in the case study of ligase annotations below). Genes identified via these erroneous annotations are not included in the final list of enzymes of a given class. Conversely, genes whose annotations are judged to be valid are added to the final list. In these cases, a new FlyBase-assigned GO annotation is made to further support the asserted activity, attributed to any newly found literature or inferred from sequence similarity as appropriate.

Next, enzyme-encoding genes identified via EC annotations or orthology to human enzymes are considered. These fall into one of the following three categories: (i) genes already identified via verified GO annotations, (ii) genes already identified (and discarded) via erroneous GO annotations or (iii) new genes not identified via GO annotations. The source/evidence for the relevant annotation(s) associated with new genes are checked—candidates with good supporting evidence added to the final list of enzymes and any additional false positive are discarded. Again, new FlyBase-assigned GO annotations are made to support verified enzymatic activities as appropriate, while annotations leading to false positives are corrected or queried in order to fix underlying errors. Examples of reasons for EC-based false positives are database asynchrony, human error in manual annotations and incorrect inferences in electronic annotation pipelines. Reasons for orthology-based false positives include erroneous GO/EC annotations to the human gene and cases where the *D. melanogaster* ortholog has been demonstrated to lack the activity shown by the human enzyme. (See the ligase case study below for further discussion of false positives.)

**Table 2:** Summary of changes in *D. melanogaster* enzyme annotations in FlyBase

**Enzyme class** **(EC number)**	**GO term (ID)**	**Number of genes**		**Genes added/removed**	**GO annotations added/removed**
		**Before analysis** ^**a**^	**After analysis** ^**b**^		
Oxidoreductases (1.-.-.-)	Oxidoreductase activity (GO:0016491)	616	649	72/39	90/13
Lyases (4.-.-.-)	Lyase activity (GO:0016829)	121	130	23/14	14/8
Isomerases (5.-.-.-)	Isomerase activity (GO:0016853)	97	104	13/6	20/2
Ligases (6.-.-.-)	Ligase activity (GO:0016874)	112	121	27/18	26/13

a ^a^Number of genes with respective GO annotations in FB2017_05 release.

b ^b^Number of genes in respective Gene Group reports in FB2018_06 release.

Additional sources of information may be relevant and available for certain enzyme subsets. For example, some enzymes are characterized by a particular protein signature and a search based on that feature can be productive (e.g. InterPro IPR002213 is diagnostic for UDP-glycosyltransferases), uncurated research papers may exist and can be found with targeted literature searches (see the case study of ligases below) or specialist online databases may be available (e.g. the CAZy database collates data on carbohydrate-active enzymes) (27).

### Verified enzyme sets

Genes categorized as true positives for a given enzyme class formed the final list of `verified’ members for that class. To date, we have reviewed four major enzyme classes: oxidoreductases, lyases, isomerases and ligases. Table 2 shows the comparison of genes annotated to these classes before and after our review, highlighting the number of genes and GO annotations added/removed in each case. Overall, we have added 135 new enzyme-encoding genes to the FlyBase annotation set and removed 77, which are supported by the creation of 150 new GO annotations and the removal of 36 erroneous annotations. These statistics demonstrate a considerable improvement in both the quantity and quality of enzyme annotations across the reviewed classes.

**Figure 2:**
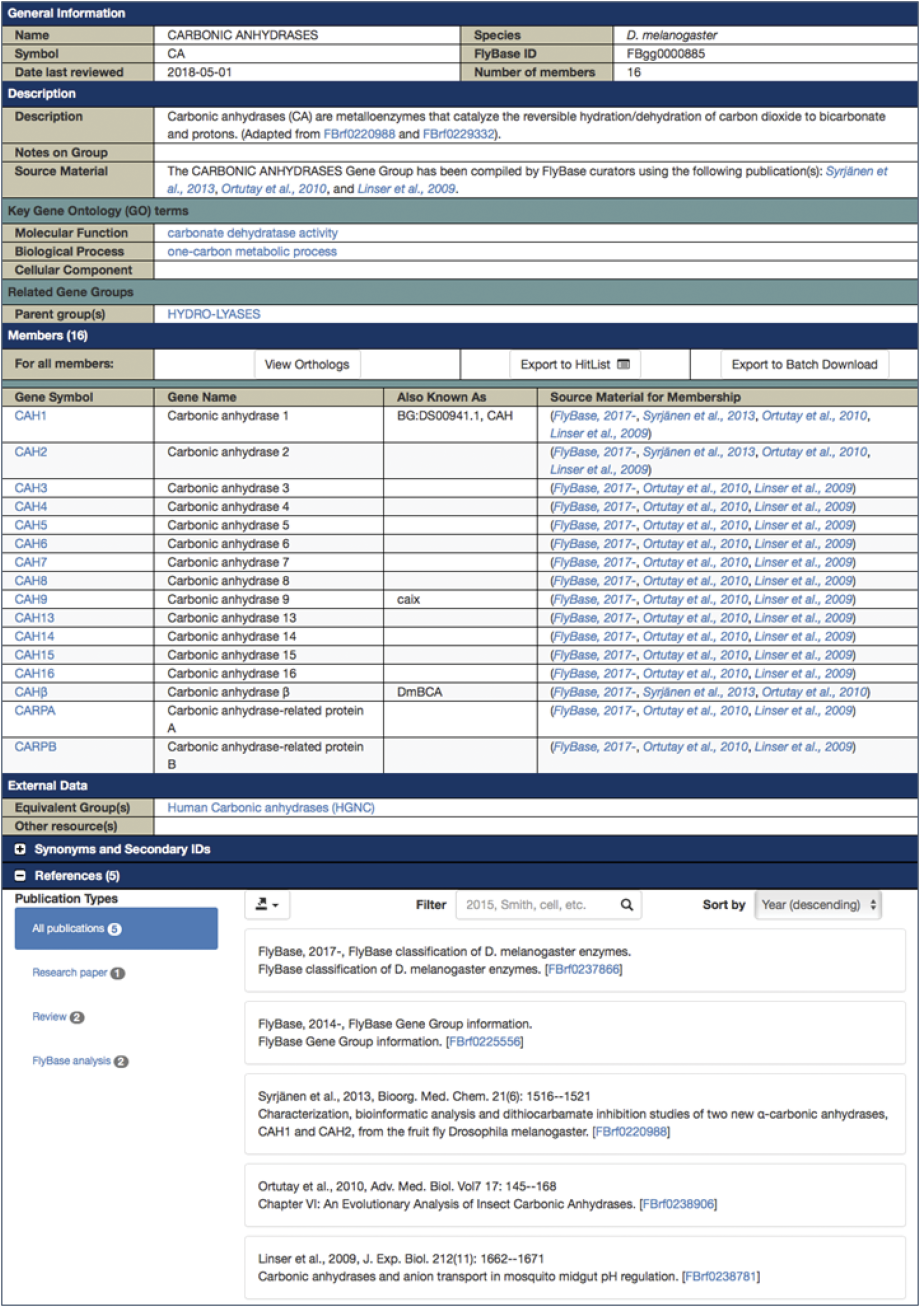
FlyBase Gene Group report for *D. melanogaster* CARBONIC ANHYDRASES, which is a subgroup of the LYASES.

**Figure 3:**
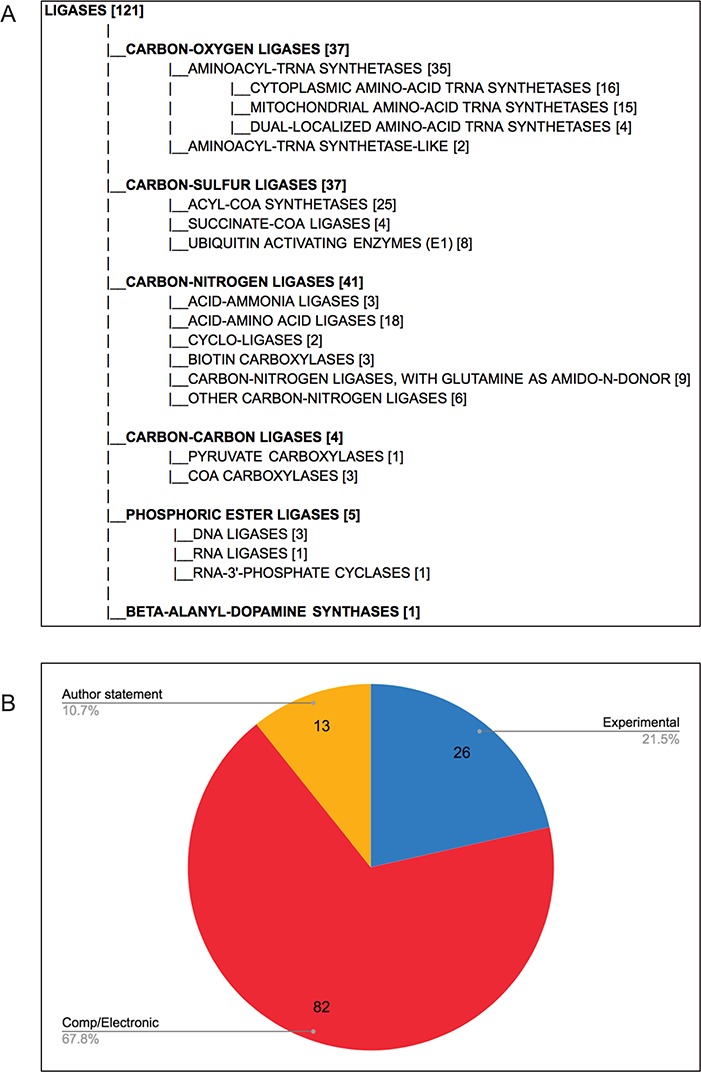
*D. melanogaster* ligases, following comprehensive review. (A) Hierarchical view of the final LIGASES Gene Group, showing the number of members in each subgroup. Note that four genes encode enzymes that fall into two different subgroups, meaning the sum of the genes in the subgroups totals 125 rather than 121. (B) Types of evidence supporting annotation as ligases in FlyBase. Experimental: ligase activity has been directly shown for the *D. melanogaster* enzyme; comp/electronic: there is no experimental evidence for ligase activity, but activity is inferred by sequence similarity, ancestry to a proven ligase and/or presence of a conserved protein signature associated with that activity (InterPro2GO mapping); author statement: there is no experimental, computational or electronic annotation evidence for ligase activity, but activity is asserted in a published research paper.

Each final enzyme list has been compiled into a Gene Group report in FlyBase (21, 28). These reports provide direct, easy access to the complete list of verified enzymes for a given class and include links to other useful FlyBase tools, the references used to compile the group and relevant external resources (Figure 2). Groups are arranged in a hierarchical fashion, based on the structure of the GO, to allow users to drill down to specific subsets. The organization of the final LIGASES Gene Group is shown in Figure 3A as an example.

The production of the final lists of verified enzymes also identified the genes that were missed from the individual sources of enzyme data (i.e. false negatives). The causes of these false negatives were explored and corrected/ticketed as appropriate. Reasons for false negatives include unidentified/uncurated literature (affecting all sources), incorrect relationships in the GO (affecting GO queries) and the absence of a clear human ortholog (affecting orthology-based searches). These and other reasons for false negatives are further examined in the case study of ligases below.

### Case study: *D. melanogaster* ligases

Ligases catalyze the joining of two molecules, or two groups within a single molecule, by forming a new chemical bond, with the concomitant hydrolysis of the diphosphate bond in ATP or a similar triphosphate (EC definition). These were the first set of enzymes we chose to review, meaning that the workflow we used incorporated additional data sources and later evolved into the more streamlined version described above. We began by collating *D. melanogaster* genes/proteins with an existing `ligase’ annotation from any of four different sources (see Materials and methods for details): (i) GO annotation to `ligase activity’ (GO:0016874) or one of its children in FlyBase; (ii) GO annotation to `ligase activity’ or one of its children in QuickGO, with no restriction on the source of or evidence for the annotation; (iii) EC annotation to EC 6 or a more specific designation at NCBI Gene or (iv) EC annotation to EC 6 or a more specific designation at UniProtKB, including both the reviewed (Swiss-Prot) and unreviewed (TrEMBL) accessions (Table 3).

**Table 3:** Analysis of original *D. melanogaster* ligase searches

	**Number of `ligase genes’ identified in given source** ^a^						
	**GO annotations**		**EC annotations**		**Orthologs of human ligases**	**New literature**	**Total unique**
	**FlyBase**	**QuickGO**	**NCBI**	**UniProt**			
**Query results**	112	138	95	106	116	10	167
**True positives**	94	101	82	89	104	10	121
**False positives**	18	37	13	17	12	n/a	46
**False negatives**	27	20	39	32	17	n/a	n/a
**Precision**	0.84	0.73	0.86	0.84	0.90	n/a	n/a
**Recall**	0.78	0.83	0.68	0.74	0.86	n/a	n/a
**F1 score**	0.81	0.78	0.76	0.78	0.87	n/a	n/a

a ^a^Using FlyBase release FB2017_05 (October 2017), QuickGO on 16 November 2017, NCBI on 20 November 2017, UniProt release 2017_10. The Orthology search used QuickGO on 23 February 2018, UniProt release 2018_01 and DIOPT version 7.0.

**Figure 4:**
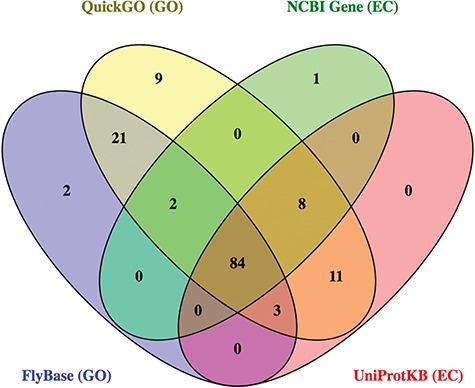
Overlap of *D. melanogaster* ligase hits from original searches.

Together, these 4 databases identified 141 potential ligases. While most hits (60%) were found in all sources, there were also significant differences between them (Figure 4). For example, 9 candidate ligases were identified only via QuickGO, and 32 candidate ligases were identified in GO-based searches that were not found using EC-based approaches. As expected, the ligases identified in the FlyBase GO search were mainly a subset of the hits from the QuickGO search, reflecting the fact that FlyBase imports a subset of all the GO annotations included at QuickGO.

Following critical review of all the GO- and EC-based annotations, a total of 40 candidates (28%) were discarded. These false positives were found across every source, though as indicated by its relatively low precision score, they were most prevalent in the annotations obtained from QuickGO (Table 3; discussed below). Together, the GO-based searches identified 101 unique true positives and 39 unique false positives, while the EC-based searches found 91 unique true positives and 18 unique false positives. Combined, these annotations identified 101 unique true positives, indicating that the EC-based searches did not contribute any new useful information above that found by GO annotations for this enzyme set. Of these 101 true positives, 24 were classed as `high confidence’ ligases, 36 as `medium confidence’, and 41 as `low confidence’, based on the quantity and type of evidence supporting their associated GO annotations (see Materials and methods).

Next, we searched for additional potential *D. melanogaster* ligases based on unambiguous orthology to characterized human ligases (see Materials and methods). This approach identified 10 novel ligases that previously lacked any GO/EC annotation to ligase activity. All have DIOPT scores of at least 8, with most scoring >10. We made a new manual GO annotation, inferred from sequence similarity, in each case so that these ligases would be found using GO searches in future. The identification of several conserved ligases via an orthology search justified the inclusion of this approach in our workflow. Indeed, this strategy had the highest precision and recall of all our original ligase searches (Table 3), reflecting both the high evolutionary conservation of this class of enzymes and the high coverage and quality of human GO/EC annotations. Twelve false positives were found using this approach, though all were caused by erroneous functional annotations to the human genes rather than inappropriate orthology calls (discussed below). Of note, all of the ligases classed as `medium confidence’ and 34 of the 41 (85%) of the `low confidence’ set were identified via the orthology-based search with a DIOPT score of at least 5 (with the majority scoring >10), thereby further supporting these inferences.

**Table 4:** Analysis of false positive *D. melanogaster* ligase annotations

**Issue**	**No. of genes**	**Sources affected**	**Action taken** ^**a**^	**Status**
Erroneous manual GO annotations	15	FlyBase, QuickGO, NCBI, UniProtKB, Orthology	Dispute/fix annotations	Done
Incorrect GO annotations via UniProtKB-Keywords2GO mapping (KW-0436)	10	QuickGO, Orthology	Helpmail to UniProt	In progress
Erroneous manual EC/keyword annotations in Swiss-Prot record	7	QuickGO, UniProtKB	Helpmail to UniProt	Done
Regular database asynchrony	6	QuickGO, NCBI, UniProtKB	n/a	n/a
Incorrect computational analysis GO annotations via the Phylogenetic Annotation and INference Tool	4	FlyBase, QuickGO, Orthology	Make tickets	Done
Incorrect GO annotations via InterPro2GO mapping	2	FlyBase, QuickGO, NCBI, UniProtKB	(Known issue)	Done
A 3-year delay in updating 2R accession in ENA from GenBank	2	QuickGO, UniProtKB	Helpmail to ENA	Done
Incorrect EC numbers submitted to INSDC by FlyBase	2	QuickGO, NCBI, UniProtKB	Fix algorithm	In progress
Incorrect relationships in the GO	1	FlyBase	(Known issue)	Done
Incorrect EC number in third-party INSDC submission	1	UniProtKB	Contact submitter	In progress
GO annotations associated with an unlocalized/orphan gene	1	FlyBase	Merge gene record	Done

a ^a^Actions marked as `n/a’ indicate that there was no practical action that could be taken; actions marked as `(Known issue)’ indicate that a fix was underway but the issue was still affecting the given sources at the time of our query.

The GO/EC-based search identified four members of the `Tubulin tyrosine ligase-like’ (TTLL) family, and the orthology-based search revealed another six members. These findings led to the discovery of an uncurated research paper that identified all 11 *D. melanogaster* TTLL genes (29), including one additional gene that had not been found by any other method. Similarly, the GO/EC search identified eight fatty acid-CoA ligases (also called acyl-CoA synthetases), and the orthology-based search revealed another four. In this case, a search for relevant literature led to two uncurated papers (30, 31) that identified a further nine fatty acid-CoA ligases. In all these cases, the relevant GO annotations were made, attributed to the given publications.

As alluded to above, a substantial number of false positive ligase annotations were found across all sources examined (Table 3). The root cause of each problem was investigated and several different types of issue were uncovered (Table 4). Some issues affected all annotation pipelines (e.g. erroneous manual GO annotations, database asynchrony) whereas others had more restricted consequences (e.g. the effects of a 3-year delay in updating a major chromosome accession in ENA were restricted to QuickGO and UniProtKB). Many false positives were associated with the historical misclassification (and/or misnaming) of enzymes that catalyze the transfer of ubiquitin to a substrate protein as `ligases’. This issue affected 7 manually reviewed UniProtKB/Swiss-Prot records and 10 unreviewed UniProtKB/TrEMBL records, with repercussions on the data obtained via our QuickGO, UniProt and orthology pipelines. Another problem was uncovered in the GO-to-EC mapping algorithm employed at FlyBase when making its annual submission to GenBank, resulting in two incorrect gene-to-EC associations. Where possible, we have implemented or initiated an appropriate remedy for the underlying issues causing false positives (Table 4).

**Table 5:** Analysis of false negative *D. melanogaster* ligase annotations

**Issue**	**No. of genes**	**Sources affected**	**Action taken** ^a^	**Status**
Incorrect relationships in the GO	38^b^	FlyBase, QuickGO	GitHub tickets	Done
Uncurated literature	23	FlyBase, QuickGO, NCBI, UniProtKB	Make annotations	Done
*D. melanogaster* gene lacks clear human ortholog	13	FlyBase, QuickGO, NCBI, UniProtKB, Orthology	Seek relevant literature	Done
Lack of an EC number equivalent to a GO term	8	NCBI, UniProtKB	Request EC term	In progress
GO annotation pipeline not used in source	4	FlyBase, NCBI	n/a	n/a
Database asynchrony	3	NCBI, UniProtKB	n/a	n/a
Only source for annotation was via orthology	2	FlyBase, QuickGO, NCBI, UniProtKB	Make ISS annotations	Done
UniProtKB/Swiss-Prot entry lacks manual EC annotation	1	UniProtKB	Helpmail to UniProt	In progress
Human ortholog lacks GO/EC annotation	1	Orthology	Helpmail to UniProt	In progress

a ^a^Actions marked as `n\a’ indicate that there was no practical action that could be taken.

b ^b^For a short time, the term `aminoacyl-tRNA ligase activity’ (GO:0004812) lacked any connection to the parent term `ligase activity’ (GO:0016874), meaning that 37 *D. melanogaster* ligase-encoding genes were not found in a QuickGO search with the parent term. This version of the GO was never imported into FlyBase owing to our update schedule, so did not impact on FlyBase searches.

**Table 6:** Analysis of *D. melanogaster* ligase searches after comprehensive annotation

	**Number of `ligase genes’ identified in given source** ^a^					
	**GO annotations**		**EC annotations**		**Orthologs of human ligases**	**Total unique**
	**FlyBase**	**QuickGO**	**NCBI**	**UniProt**		
**Query results**	121	142	98	95	111	151
**True positives**	121	121	87	86	103	121
**False positives**	0	21	11	9	8	29
**False negatives**	0	0	34	35	18	n/a
**Precision**	1.00	0.85	0.89	0.91	0.93	n/a
**Recall**	1.00	1.00	0.72	0.71	0.85	n/a
**F1 score**	1.00	0.92	0.79	0.80	0.89	n/a

a ^a^Using FlyBase release FB2018_05 (October 2018), QuickGO on 18 October 2018, NCBI on 18 October 2018, UniProt release 2018_09. The Orthology search used QuickGO on 18 October 2018, UniProt release 2018_09, and DIOPT version 7.1.

Overall, we validated a total of 121 *D. melanogaster* ligases (Figure 3A), the majority of which are supported by computational analysis or electronic annotations rather than direct experimental evidence (Figure 3B). This final number allowed us to assess the false negatives rate in the five sources we used (Table 3). GO-based searches performed better than EC-based searches, as reflected in their respective recall scores, indicating that GO annotation provides better coverage of enzyme annotation compared to EC annotation, at least for this data set. We also probed the reasons why certain ligases were missed in our original sources (Table 5). As observed for false positives, the underlying explanations for false negatives are various and affect the sources differently. The two major reasons were changes to relationships within the GO itself (that affected the number of hits obtained from a query with the parent `ligase activity’ term), and uncurated functional information in the published literature. Other reasons included the lack of an EC cross-reference to a GO term (which affected EC-based queries) and the absence of a clear human ortholog (which primarily affected the orthology-based approach to identifying ligases).

At the start of this work, a GO-based search in FlyBase (FB2017_05) for genes encoding *D. melanogaster* ligases gave 112 hits. We now know that 18 of those were incorrect, while 27 ligases with good supporting evidence were missing. The same search today (FB2018_05) retrieves all 121 validated ligases with no false negatives (Table 6). Moreover, the FlyBase Gene Group page for the LIGASES collates all these genes into a single report (http://flybase.org/reports/FBgg0000801). Improvements in precision and recall measures for a `ligase’ query are also evident for the other databases/pipelines employed in this work (compare Table 6 to 3)—this is particularly noticeable for the QuickGO query, while improvements to the EC-based searches will require more time for updates to filter through to NCBI Gene and UniProtKB-TrEMBL.

## Discussion

This paper describes our ongoing approach to systematically review and enhance the functional annotation of *D. melanogaster* enzymes. Our primary aim is to provide full and accurate enzymatic data within FlyBase but, by utilizing the GO as our main annotation medium, our work also benefits users accessing *D. melanogaster* GO annotations through third-party sites such as QuickGO, AmiGO, UniProtKB or the recently formed Alliance of Genome Resources (32). The summary statistics presented for the four enzyme groups reviewed to date (Table 2) demonstrate the extent of the improvements and the effectiveness of our strategy. An important finding was that no single database/search strategy provided the full enzyme set prior to our review—data from several sources needed to be synthesized and integrated to generate a comprehensive set, and many of the errors/omissions we uncovered would have been missed using more restricted or piecemeal approaches. The same general workflow could be employed to improve similar data sets at other databases.

Our case study on ligase annotations clearly showed how an apparently identical query can generate very different results from different databases and that the reasons for the discrepancies (and therefore which results might be `trusted’) are not immediately obvious—even to a biocurator! The two major reasons for these differences were determined to be database asynchrony and the different policies/pipelines implemented in different resources for showing functional annotations. For example, the majority of *D. melanogaster* EC data in NCBI and UniProtKB/TrEMBL are based on FlyBase GO annotations (via the GO-to-EC mapping FlyBase computes in its annual submission to GenBank), but may be a year or more out of sync with current GO data; QuickGO includes UniProtKB electronic GO annotation pipelines that are not present in FlyBase. Although these explanations are reasonable and, to some extent, unavoidable, all databases should make more effort to clearly document and display the provenance of the GO/EC data they display, including the version/date of any third-party data they import. That said, there is also a need to educate database users that any single resource may not provide a complete and accurate search result. For example, we determined that our initial query of QuickGO for *D. melanogaster* ligases had a precision and recall of 0.73 and 0.83, respectively, indicating a significant number of erroneous and missing entries. Thus, multiple sources of data may need to be consulted, and an integrative approach that accounts for the evidence/source of the data may be required to obtain a full and reliable answer to these types of query.

It is notable that only a fifth of the `verified’ ligases have direct experimental evidence for having ligase activity in *D. melanogaster* (Figure 3B). Rather, the majority are only inferred to have this activity based on conservation of sequence, biological ancestry and/or presence of a protein domain that is associated with that activity. On the one hand, this example illustrates the power of modern-day bioinformatics—knowledge established for one or a family of gene products can be reasonably transferred to other related gene products. Indeed, such inferences are well established within the framework of GO annotation (24, 33) and are distinguished by a distinct set of evidence codes (12). However, such predictive annotations must be treated with due diligence by researchers and verified where possible.

This project also highlighted the need for a better source of EC annotations to *D. melanogaster* enzymes. To this end, FlyBase will soon display EC numbers, names and reactions on its Gene Reports. These data will be computed from the current set of GO annotation data with each FlyBase release by utilizing the EC cross-references within the Molecular Function aspect of the GO. This approach means that FlyBase EC annotations will be fully automated and kept in sync with current GO data, while allowing curators to control the EC annotations via updates to GO annotations and/or requesting EC cross-reference updates within the GO. Moreover, this strategy means the evidence code present within GO annotations can be used to distinguish EC annotations based on experimental evidence from those based on predictions.

Importantly, our work results in improvements in functional annotation beyond just *D. melanogaster*, in at least two different ways. First, many of the false positive and false negatives annotations we observed were caused by errors/omissions within the GO itself or in centralized computational or electronic GO annotation pipelines. By alerting the responsible parties and fixing the underlying issues at source, we are improving the accuracy of enzyme-related GO annotations/queries for all species. Second, our work will benefit the functional annotation of non-model insect/arthropod genomes because the improved GO annotations for *D. melanogaster* can be transferred to these related species.

To date, we have reviewed four major enzyme classes (oxidoreductases, lyases, isomerases and ligases), comprising around 1000 (25%) of the approximately 4150 enzyme-encoding genes in *D. melanogaster*. We consider these sets, as represented by FlyBase Gene Group reports, to be accurate and complete based on current data, though they will need to be re-assessed every few years to ensure that new data are incorporated. We also hope to complete the review of the remaining major enzyme classes (transferases, hydrolases, translocases) in the coming years, continuing to work with other biocurators and databases in order to benefit fly researchers and the wider biological community.

## Authors’ contributions

P.V.G., J.Z., A.J.R. and S.J.M. performed the analysis. P.V.G., A.J.R. and S.J.M. edited/created GO annotations and reported errors to the responsible parties. P.V.G and S.J.M. wrote the paper. S.J.M. conceived and supervised the project.

## Notes

**Database URL:** www.flybase.org
